# Material Flows and Greenhouse Gas Emissions Reduction Potential of Decentralized Composting in Sub-Saharan Africa: A Case Study in Tiassalé, Côte d’Ivoire

**DOI:** 10.3390/ijerph17197229

**Published:** 2020-10-02

**Authors:** Dotanhan Yeo, Kouassi Dongo, Adeline Mertenat, Phillipp Lüssenhop, Ina Körner, Christian Zurbrügg

**Affiliations:** 1 Unité de Formation et de Recherche des Sciences de la Terre et des Ressources Minières (UFR STRM), Université Félix Houphouët-Boigny, 22 BP 582 Abidjan 22, Cote d’Ivoire; kouassi.dongo@csrs.ci; 2 CSRS: Centre Suisse de Recherches Scientifiques en Côte d’Ivoire, 01 BP 1303 Abidjan 01, Cote d’Ivoire; 3 Eawag: Swiss Federal Institute of Aquatic Science and Technology, 8600 Dübendorf, Switzerland; adeline.mertenat@eawag.ch (A.M.); zurbrugg@eawag.ch (C.Z.); 4 Institute of Wastewater Management and Water Protection, Bioresource Management Group, Hamburg University of Technology, Eissendorfer Str. 42, 21073 Hamburg, Germany; phillipp.luessenhop@tuhh.de (P.L.); i.koerner@tuhh.de (I.K.)

**Keywords:** sub-Saharan Africa, decentralized composting, material flow, GHG emissions

## Abstract

Despite many composting initiatives implemented in recent years throughout Sub-Saharan Africa, there is yet a lack of data on material flows and the potential contribution of decentralized composting towards greenhouse gas (GHG) mitigation. This study fills this gap assessing flows, emissions reduction and other environmental benefits of decentralized composting, based on a pilot composting facility implemented in the municipality of Tiassalé in Côte d’Ivoire. Primary data collected at the site were visualized with the STAN version 2.6 software developed at the Vienna University of Technology (Austria), for material flows, while carbon emissions reduction was estimated using the UNFCCC methods. Results show that in 2017, from the 59.4 metric tons of organic waste processed by this pilot station, 14.2 metric tons of mature compost was produced, which correspond to 24% of the input mass (on wet weight basis). On dry weight basis, mature compost represents 36% of the input mass. The nutrient content of the compost is in line with data from literature on sub-Saharan African compost, and heavy metal contamination fulfils both French and German compost standards. Concerning the GHG emissions reduction potential, the results show that with this composting scenario, 87% of the baseline emissions occurring in open dumping can be avoided.

## 1. Introduction

In the last decade, urbanization and population growth led to a significant increase of waste generation [1,2,3]. The collection, transport and processing of these growing quantities remain a major problem in many countries, especially in low- and middle-income countries where waste collection rate is generally below 50% [4,5].

The consequences of this poor management are twofold; on one hand, uncollected waste accumulates in drains, on roads and open public spaces, contributing to floods, air pollution and the spread of disease vectors [5,6]. On the other hand, the collected waste is dumped in unmanaged landfills, polluting soil and fresh water by leachates and producing methane (CH_4_), a greenhouse gas 28 times more powerful than CO_2_ [7]. CH_4_ is generated from anaerobic decomposition of organic matter. The organic matter accounts for between 50% and 70% of municipal solid waste stream in low- and middle-income countries [8,9].

The organic fraction of municipal solid waste consists of a large amount of easily biodegradable substances such as food wastes and therefore can be composted [10]. The advantages of composting are multiple: It extends landfill lifetime, reduces greenhouse gas emissions and, above all, produces a hygienic organic fertilizer and soil improver (compost), which makes it particularly suitable for ensuring carbon sequestration in soil and soil fertility in low- and middle-income countries’ contexts. Large-scale composting plants are widespread in developed countries, but in low- and middle-income countries, with failure of large-scale composting plants, NGOs and researchers are now promoting decentralized composting as an alternative [11]. It has been reported that the failure of dealing with centralized composting plants in low- and middle-income countries was attributed to several factors including high transportation costs, high investment and operation costs, inappropriate technologies as well as low quality of the produced composts. In contrast to these large scale composting plants, decentralized composting is less expensive, requires less qualified skills, and in low- and middle-income countries’ results in higher quality composts [12].

In sub-Saharan Africa, organic waste composting, however, remains proven only at a pilot scale [13,14]. The effective integration of decentralized composting into municipalities’ waste management strategies could better be achieved if decision-makers had a better understanding of the compost benefits, material flows, economic viability and environmental benefits of these units. Few previous studies on composting in sub-Saharan Africa have focused on the assessment of compost quality [15,16,17] and some on the economic viability of decentralized composting plants [18,19]. However, evidence of the environmental benefits considering the material flows analysis (MFA) and the potential of GHG emissions reduction is still limited.

Material flow analysis is a systematic assessment of the flows of materials within a defined space and a certain time. This tool is based on the mass balance principle [20,21]. MFA is widely used in waste management field for environmental impact assessment of waste treatment plants as well as for plants performance and efficiency evaluation [22,23]. For example, Jensen et al. [24] performed a MFA to obtain the transfer coefficients for a combined dry anaerobic digestion and post-composting facility in Spain. Guo et al. [25] studied the material flow on dry weight basis of a food waste (FW) bioconversion plant using black soldier fly larvae (BSFL) in China. In addition, Padeyanda et al. [26] used MFA to assess the environmental impacts of the different management stages (collection and transportation, treatment, and material use) of food waste in South Korea.

Several studies [27,28,29,30] have shown that organic waste composting results in important GHG savings. These savings depend on waste type and composition (kitchen organics, garden waste), technology type (open systems, closed systems, home composting), equipment used at the composting plant (shredder, compost turner, Front loader, tractor), and the use of compost [31]. However, few studies have tackled GHG savings issues related to decentralized composting plant.

This study aimed at assessing the material flows and GHG emissions reduction potential of a pilot decentralized composting plant in Côte d’Ivoire, treating source-separated organic waste. The functional unit mentioned in this study was 60 tons of organic waste treated by the pilot composting plant during a year (2018), and the GHG emissions reductions were estimated using the United Nations Framework Convention on Climate Change (UNFCCC/CCNUCC) methodological tools for clean development project (CDM). This method considers only methane and nitrous oxide emissions. The importance of the conduct MFA is due to the fact that these data could be used to optimize the composting process and provide a baseline for designing future units. The information on GHG emissions reduction by decentralized composting plants could encourage central governments to promote decentralized composting as a way of meeting their nationally determined contributions (NDCs) in mitigating greenhouse gas emissions agreed at the COP 21 in Paris. Furthermore, these data could be used as background information for LCA in Sub-Saharan African countries contexts.

## 2. Materials and Methods

### 2.1. Description of the Composting Plant and Process

**Figure 1:**
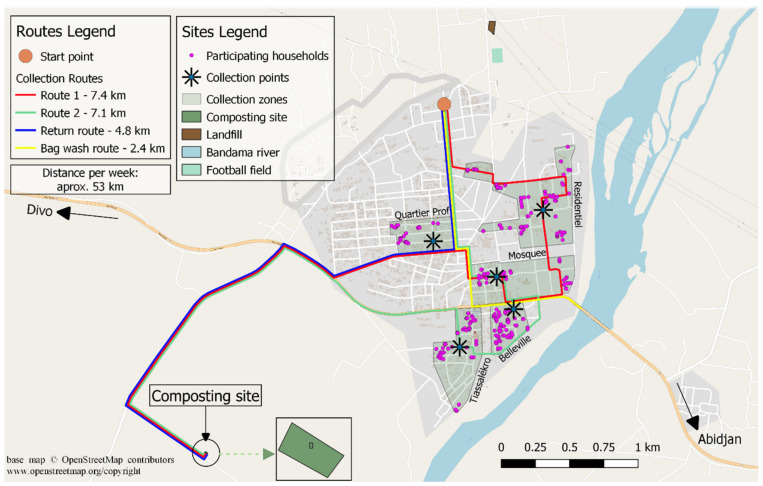
Waste collection map: collection points and routes in Tiassalé.

**Figure 2:**
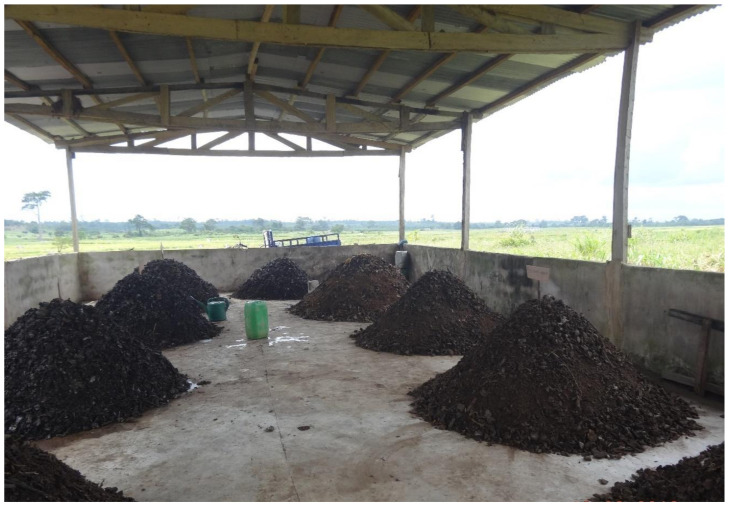
Composting plant: pilot tests in Tiassalé.

This study focused on a pilot decentralized composting plant in Tiassalé, a city of 20,057 inhabitants located about 130 km from Abidjan, the economic capital city of Ivory Coast. The composting unit processes source separated municipal solid waste (SSMSW) collected from 230 households (Figure 1) with an average household size of 8. The compositing unit has a full treatment capacity of 100 metric tons per year. The organic waste composted was mainly composed of cassava skin, plantain banana skin and other kitchen waste. The composting plant has a roof to avoid rainfall saturating the waste windrows leading to nutrient loss and a solid underground, which prevents leachate from reaching the groundwater. It is further surrounded by a fence of 1 m height, to prevent access to animals (Figure 2).

The organic waste was collected three times a week using a moto trike. Waste collected within a week (about 1.5 metric ton) was weighed after the third collection day. During the weighing process, a few inorganic fractions still present in waste, despite the source-separation (e.g., plastic bags, diapers, etc.), were sorted manually, and then, the waste was heaped in windrows of 2 m diameter and 1.5 m height. The waste mixture had a bulky structure enabling penetration of air from the surroundings. In this naturally ventilated system, the so-called chimney effect caused a certain air circulation. The composting process took 3 months: 1 month for the active composting phase and 2 months for compost maturation. During the active phase, the windrows were turned and watered with about 40 litters of water once a week. At the end of the 3 months, the mature compost was sieved into three size fractions (Φ): the residues (Φ > 20 mm) and two classes of composts (20 < Φ < 10 mm and 10 mm < Φ). The residues were transported to the landfill, whereas the other two compost classes went into the commercial market for local agriculture.

### 2.2. Sampling and Waste Characterisation

Two waste characterization campaigns of one week each were performed at the composting plant. The first one was carried out during the raining season, and the second one during the dry season in order to consider the seasonal variation. For each campaign, waste collected during a week was weighted, homogenized and reduced by quartering method [32]. Fresh waste was divided randomly in 4 equal parts, then two opposite parts were rejected, and the two others were mixed and divided again into 4 parts. The same procedure was repeated until a sample size of about 125 kg was obtained as recommended by the French MODECOM^™^ [33]. This sample was then sorted into 6 different types of waste: kitchen waste; garden waste; paper and cardboard; wood; textiles; and inorganic substances such as plastics, stones, etc. Each category was then weighed separately.

### 2.3. Analytical Measurements

Two sampling campaigns were performed to determine the physical-chemical characteristics of the input and output materials. For each campaign, five samples of the input materials were extracted from different windrows. These samples were mixed and then divided into two parts of 1 kg each. The first part was sent directly to the laboratory for analysis of total solids (TS) and volatile solids (VS). The second part was sun dried, then shredded and stored in the laboratory for later conduction of physical-chemical analysis. The same procedure was performed for the mature compost regarding the outputs sampling.

Total solids (TS) contents of the samples was measured by drying the samples at 105 °C for 24 h. Volatile Solids (VS) contents was measured as mass loss after heating the dry sample at 550 °C for 2 h [34]. Electrical conductivity and pH were analyzed in a slurry of compost and deionized water in a mass ratio of 1:5. The carbon content was estimated from volatile solid content (Equation (1)), using 1.8 as VS/C ratio [35].

(1) $$\%C=\frac{\left(100-\%Ash\right)}{1.8}$$

The total nitrogen (TKN) was measured using the Kjeldahl method described by [36], and P, K, Cd, Cr, Ni, Pb, Cu and Zn were measured using ICP-OES method [34].

### 2.4. System Boundaries and Material Flow Analysis

**Figure 3:**
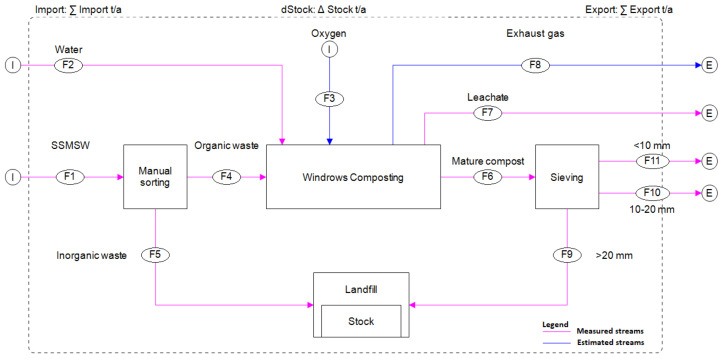
Boundary of the material flow analysis.

The system boundary for the material flow analysis is shown in Figure 3. It includes the composting plant and the municipal landfill. Waste collection and transport are however excluded. They were assumed not to influence the material flow. During the study, input and output weights, leachate amounts and addition of water volume were measured and noted.

The composting process was not actively aerated; however, oxygen (O_2)_ entered the composting windrows by passive aeration processes.

The amount of oxygen consumed during the composting process has been estimated assuming that the mass loss of organic matter goes mainly back to carbohydrates with the main building block glucose. Then, it could be calculated stoichiometrically via Equation (2) using the Molar masses of glucose and O_2_ (.M_O2_: 31,998 g/mol, M_Glucos*e*_: 180,156 g/mol).

(2) $$C_{6}H_{12}O_{6}+6O_{2}\to 6H_{2}O+6{\mathrm{CO}}_{2}$$

The organic matter mass lost was calculated via the masses of organic waste and mature compost and their respective total solid (TS) and ash contents (Equation (3)), and subsequently, the O_2_ consumed was calculated via Equation (4).

(3) $$m_{\mathrm{loss},\mathrm{organic}\mathrm{matter}}\left(\mathrm{kg}\right)={\left[\left(m\left(\mathrm{kg}\right)*\mathrm{TS}\right)\left(1-\mathrm{Ash}\right)\right]}_{\mathrm{Organic}\mathrm{waste}}-{\left[\left(m\left(\mathrm{kg}\right)*\mathrm{TS}\right)\left(1-\mathrm{Ash}\right)\right]}_{\mathrm{Matured}\mathrm{compost}}$$

(TS, ash: value in %/100)

(4) $$m_{O2}\left(\mathrm{kg}\right)=\frac{m_{\mathrm{loss},\mathrm{organic}\mathrm{matter}}\left(\mathrm{kg}\right)*6\;*\;M_{O2}\left(\frac{g}{\mathrm{mol}}\right)}{M_{\mathrm{Glucose}}\left(\frac{g}{\mathrm{mol}}\right)}$$

Material flow visualization was performed using STAN version 2.6 software developed at the Vienna University of Technology (Austria) [37]. STAN was also used to estimate the mass of gaseous releases during the composting process by mass-balance. These estimations were performed through the Equation (5).

(5) $$m\left(\mathrm{exhaust}\mathrm{gas}\right)=m\left(\mathrm{measured}\mathrm{inputs}\right)+m\left(\mathrm{calculated}\mathrm{inputs}\right)-m\left(\mathrm{outputs}\right)$$

where

m (measured inputs): mass from cleaned organic waste entering the composting process and from water added during composting;

m (calculated inputs): mass from oxygen (O_2_) from surrounding air consumed by composting microorganisms;

m (outputs): mass from mature compost received at the end of composting and from leachate generated during composting.

### 2.5. Emissions Reduction Calculation

The evaluation of composting plant emissions reduction for a year of activities was performed according the United Nations Framework Convention on Climate Change (UNFCCC/CCNUCC) for clean development projects (CDM). This emissions reduction (ER) represents the quantity of GHG emissions avoided by the composting project. In other words, ER is the difference between the baseline emissions and the sum of the project’s emissions and leakages (Equation (6)).

(6) $$\mathrm{ER}={\mathrm{BE}}_{\;\mathrm{CH}4,\mathrm{SWDS},y}-\;\left({\mathrm{PE}}_{\mathrm{COMP}}\;+{\;\mathrm{LE}}_{\mathrm{COMP}}\right)$$

where

ER = Emission Reduction (t CO_2-eq_/a);

BE_CH4,SWDS,y_ = Baseline, GHG emissions occurring in year y generated from waste at a solid waste disposal site (SWDS) during a time period ending in year y (t CO_2-eq_/a);

PE_COMP_ = Project GHG emissions from the composting process in year y (t CO_2-eq_/a);

LE_COMP_ = Leakage emissions associated with composting in year y (t CO_2_e/a).

#### 2.5.1. Baseline Emissions

In this study, the baseline was the traditional situation where organic waste is disposed of at municipal landfills, and the baseline emissions represent the GHG emissions that, in the absence of the composting project, would be generated from the anaerobic decay of the organic waste disposal at the municipal landfill. In fact, anaerobic decomposition of organic waste produces significant amounts of carbon dioxide (CO_2_), methane (CH4) and non-methane volatile organic compounds (NMVOCs) and smaller quantities of nitrous oxide (N_2_O), nitrogen oxides (NO_x_) and carbon monoxide (CO). CO_2_ emissions were not included in the baseline emissions calculation since these emissions are considered as biogenic CO_2_ [38]. The methodological tool 04 “Emissions from solid waste disposal sites” version 08 was used for baseline emissions estimation. This method is based on the first order decay model (Equation (7)) developed by the Intergovernmental Panel on Climate Change [39]. Solely the methane emissions are considered in this model.

(7) $${\mathrm{BECH}}_{4,}\;\mathrm{SWDS},\;y\;=\;\varphi \;\times \;\left(1-\mathrm{fy}\right)\times {\;\mathrm{GWPCH}}_{4}\;\times \;\left(1\;-\;\mathrm{OX}\right)\times \frac{16}{12}\times \;F\;\times \;\mathrm{DOCf},\;y\;\times \;\mathrm{MCFy}\;\;\times \;\Sigma \Sigma \mathrm{Wj},x\;\times \;\mathrm{DOCj}\;\times {\;e}^{-\mathrm{kj}}\times \;\left(y-x\right)\times \;\left(1-e^{\left(\mathrm{kj}\right)}\right)$$

where

φ = Model correction factor to account for model uncertainties for year y;

fy = Fraction of methane captured at the SWDS and flared, combusted or used in another manner that prevents the emissions of methane to the atmosphere in the year y;

GWPCH_4_ = Global Warming Potential of methane;

OX = Oxidation factor (reflecting the amount of methane from SWDS that is oxidized in the soil or other material covering the waste);

F = Fraction of methane in the SWDS gas (volume fraction);

X = Years in the time period in which waste is disposed at the SWDS, extending from the first year in the time period (x = 1) to year y (x = y);

Y = Year of the crediting period for which methane emissions are calculated (y is a consecutive period of 12 months);

DOCf,y = Fraction of degradable organic carbon (DOC) that decomposes under the specific conditions occurring in the SWDS for year y (weight fraction);

MCFy = Methane correction factor for year y;

Wj,x = Amount of solid waste type j disposed or prevented from disposal in the SWDS in the year x (t);

DOCj = Fraction of degradable organic carbon in the waste type j (weight fraction);

Kj = Decay rate for the waste type j (1/a);

j =Type of organic waste in the MSW.

As it can be seen in this equation, the amount of CH_4_ generated depends on organic waste quantity, composition and characteristics and the dumpsite characteristics. Waste composition used for the calculation was based on the data of waste characterization campaigns described in Section 2.2. Concerning waste and dumpsite characteristics, the default values in the UNFCCC tool 04 “Emissions from solid waste disposal sites” version 08 were used (Table 1).

In Tiassalé, as well as in most cities of sub-Saharan Africa, landfills are not equipped with methane capture systems and not covered with aerated material, so the fraction of methane captured at the SWDS and flared, combusted (fy) and oxidation factor (OX) were set to zero.

#### 2.5.2. Project Emissions Estimation

The project emissions calculation was based on the Methodological tool 13 “Project and leakage emissions from composting” version 2. In this study, transportation emissions are not accounted for because they were assumed to be similar to those of the baseline [40]. Given the fact that the composting plant under this study is operated without electricity or fuel consumption, the project emissions were limited to the GHGs emitted during the composting process. The carbon dioxide produced during the composting process is not considered as a greenhouse gas because of it biogenic origin [41]. Therefore, only methane (CH_4_) and nitrous oxide (N_2_O) emissions were taken into account for project emissions (Equation (8)).

(8) $${\mathrm{PE}}_{\mathrm{COMP}}=Q_{y}\times {\mathrm{EFCH}}_{4,y}\times {\;\mathrm{GWPCH}}_{4}\;+Q_{y}\times {\mathrm{EFN}}_{2}O{,}_{y}\times {\mathrm{GWPN}}_{2}O\;$$

where

PE_COMP_ = Project emissions of GHGs from the composting process in year y (tCO_2_-eq/a);

Q_y_ = Quantity of waste composted in year y (t/a);

EFCH_4_,_y_ = Emission factor of methane per ton of waste composted valid for year y (tCH_4_/t);

GWPCH_4_ = Global Warming Potential of methane;

EFN_2_O_,y_ = Emission factor of nitrous oxide per ton of waste composted valid for year y (tN_2_O/t); GWPN_2_O = Global Warming Potential of N_2_O (tCO_2_-eq/tN_2_O).

These emissions were calculated using the default values of methane and nitrous oxide emissions factors (Table 2) [40]. Concerning the global warming potential of CH_4_ and N_2_O, the fifth commitment values were used [7].

#### 2.5.3. Leakage Emissions

Leakage emissions correspond to CH_4_ emissions due to anaerobic decay of compost or residues. These emissions are considered when the compost is subject to anaerobic storage. In this study, these emissions were set to zero given the fact that the compost was used as fertilizer in aerobic conditions, and the residues were used as a cover material at the municipal landfill [40].

## 3. Results and Discussion

### 3.1. Waste and Compost Quality

Table 3 presents the physico-chemical characteristics of the inputs and outputs materials. These analyses show that the input materials (wastes) are characterized by a neutral pH (6.7), this value is in the range (6.5–7.5) recommended for efficient composting [42]. Water content of inputs materials (41.6%) is below the recommended range of 50–60% water content [43], so an additional amount of water should be added during the active phase to run the composting process efficiently. The C/N ratio of inputs materials (34) was found in the recommended range (20–40) for composting [44]. This value (in upper range) is probably due to the high carbon contents in the main waste components which consist of plantain and cassava peels.

The pH of composts produced was basic pH (9.1), which is probably due to the high K^+^ content (11.4 g/kg) in the inputs’ materials, which accumulated due to organic mass loss. In fact, in its water-soluble form, K^+^ ions can react with bi-carbonic acids (^−^HCO_3_) obtained during the mineralization of organic matter to form potassium hydroxide (KOH), which increases the pH [45]. The use of these composts in agriculture could therefore reduce the acidity of the soils in this region, which have pH levels between 5 and 6 [46] and can improve their capacity to retain nutrients. The strong decline of the input C/N from 34 to a compost C/N ratio of 11.3 indicates high releases of C due to organic matter decomposition. According to Pearson et al. [47] and Confesor et al. [48] C/N rations below 15 are a hint for stability and maturity of the compost. The nutrients (NPK) content of the compost produced is in the range of values mentioned in the literature [15,16,17] [49]. These concentrations are higher than in feedstock given the loss of mass during the composting process [50].

The heavy metals content of compost is far below the threshold limits of both French and German compost norms (Table 4) and lower than values found in the literature on sub-Saharan African composts [15,16]. These results are also lower than the average values obtained by Rodrigues et al. [51] at 20 plant in Catalonia (Spain). This reflects low contamination of the inputs materials, thus confirming the observation and household’s practice and acceptance of the waste segregation system implemented in this study. The compost produced shows promising results in quality, but a study on crop growth with such compost still needs to be finalized to confirm this result.

### 3.2. Material Flows

**Figure 4:**
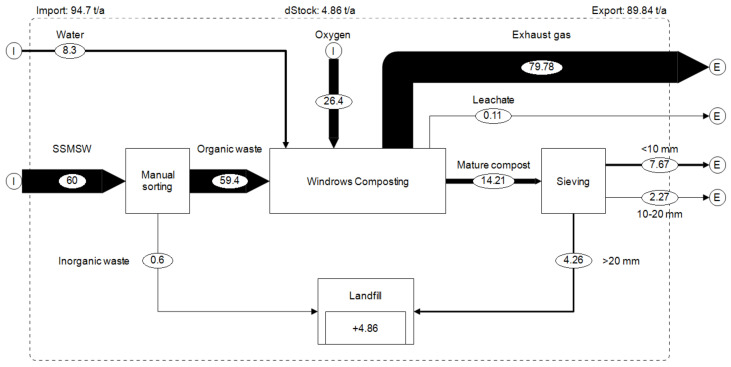
Material flow (wet weight) in the composting plant (metric ton/year).

Figure 4 shows the 2017 material flow (by wet weight) for the composting plant in Tiassalé. During the year of study, from the 60 metric tons of segregated waste collected from selected households, about 0.6 metric ton was non-biodegradable (mainly plastic) and 59.4 metric tons was organic waste.

Of these 59.4 metric tons of organic wastes composted, 14.21 metric tons of mature compost was obtained, which corresponds to 24% of the input mass (wet weight). This mature compost consists of 54% compost with a particle size of 10 mm or lower, 16% with particle size between 20 and 10 mm and 30% of residues with particle size of 20 mm or above, which was transported to the municipal landfill where it was used as cover material.

On wet weight basis, the compost production rate (24%) was quite similar to Chee and Sunami [32] (run 2) who obtained 27% of input mass after aerobic treatment of organic waste. However, this value is low compared to Andersen et al.’s [53] results which obtained between 34% and 46% of inputs mass after household organic waste and garden waste composting. This high value from Andersen et al. [53] is probably due to the high water content (67–75%) of their composts, when in our study the final moisture content was much lower (12.6%).

**Figure 5:**
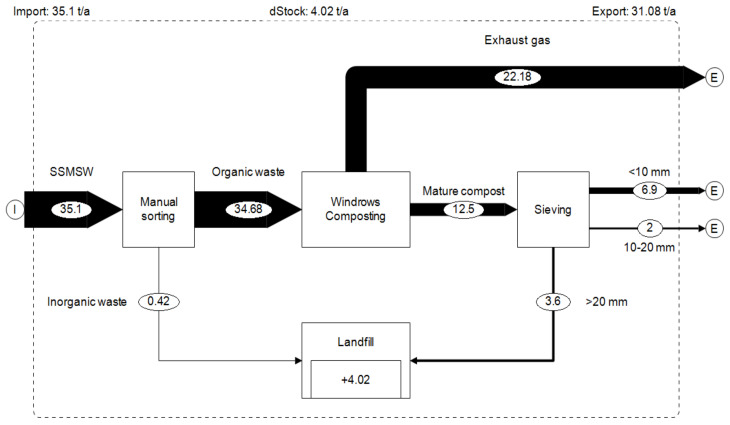
Material flow (dry weight) in the composting plant (metric ton/year).

On dry weight basis, 35.1 metric tons of biowaste (dry weight) was composted resulting in 12.5 metric tons of compost, which represents 36% of inputs mass (Figure 5). The difference in dry mass of 22.1 metric tons (64%) can be assumed lost through biological metabolism (gaseous emissions). This result is close to Andersen’s et al.’s results, who obtained an average of 40% of inputs mass (on dry weight basis). This similarity could be due to the fact that the inputs material composition of both studies is quite similar (mix of about 95% of food waste and 5% of garden waste). However, this compost production rate on dry weight basis is lower than the 51% obtained by Guo et al. [25] at a food waste bioconversion plant using black soldier fly larvae (BSFL).

**Figure 6:**
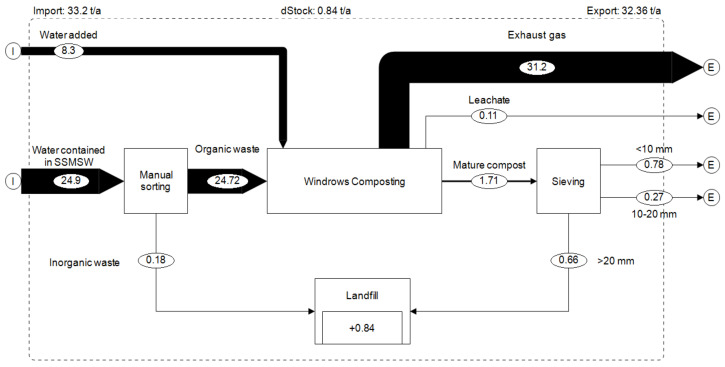
Water flow in the composting plant (cubic meters/year).

During the composting process, 8.3 cubic meters (equivalent to a metric ton) of water were used for the composting process. In other words, a composting plant with a treatment capacity of 10 metric tons of organic matter per day under similar conditions would therefore need about 504 cubic meters of water annually. Access to this significant amount of water might be problematic in arid regions of sub-Saharan Africa. Under such climatic and waste composition conditions, the composting units should ensure having a reliable water source and access, either through a bore well or another reliable water access point. As mentioned in Figure 6, a significant proportion of total water (94.5%) was emitted to the environment as water vapor, while 5.2% remained in the mature compost, and 0.3% ran off as leachate. The low water content of mature compost has the advantage of reducing the transportation cost.

### 3.3. Emissions Reduction

Waste characterization campaigns revealed that the main components of organic waste collected was food waste (82.5%) and garden waste (5.2%) as illustrated in Table 5. The percentage of impurity (inorganic materials) arriving at the composting plants was low (1%) due to the effective source segregation and door-to-door collection systems implemented in this study [54].

The baseline emission was estimated based on waste amounts and composition for a period of 21 years which is the maximum period for CDM small-scale project [28]. In this scenario, which did not have any diversion of organic waste (by composting) from the disposal site, 52.4 tCO_2_-eq (Equation (7)) originating from CH_4_ and N_2_O would be released in the atmosphere (Table 6). In other words, for every ton of organic waste dumped at the disposal site, 0.9 tCO_2_-eq are produced.

The composting project scenario also causes the emissions of 6.9 tCO_2_-eq (Equation (8)) meaning that for every ton of organic waste composted, 116 kgCO_2_-eq was emitted to the atmosphere. These emissions are however far lower and represent only 13% of the baseline scenario emissions. Therefore, in only one year of activity, this pilot composting unit has been able to reduce GHG emissions of 45.5 tCO_2_-eq (Equation (6)), or an emission reduction of 87%. Such emission reduction by composting facilities is comparable to results from literature, for instance as found in the last monitoring report from Lahore composting plant (85%) [55].

The information gathered from this investigation is relevant in the sense that it represents a pathway that will help in the estimation of the impact of decentralized organic waste composting if it would be scaled for the whole city of Tiassalé. For instance, if Tiassalé, where 30 metric ton of organic waste is generated daily, would construct and operate a total of 3 decentralized composting units with a capacity each of 10 metric ton/day (as these plants are managed manually), the city could reduce GHG emissions by 8294 tCO_2_-eq per year and produce 7.2 metric ton of compost daily.

Furthermore, the emissions reduction would be even higher if the calculation would consider the avoidance of emissions from mineral fertilizer production given its substitution by compost. For instance, Urea is reported [56] to generate 1.85 tCO_2_-eq per ton of Urea produced (containing 460 kg Nitrogen). With an average nitrogen content in compost of 1.59% (Table 3), 29 metric tons of compost could substitute the nitrogen in 1 metric ton of Urea. With a production of 7.2 metric tons of compost per day, the additional emissions reduction by avoiding urea production can be estimated at 0.4 tCO_2_-eq per day or 146 tCO_2_-eq per year.

The emissions avoided could be sold on the voluntary carbon market, which assuming only disposal site diversion and a market price of around 20 €/tCO_2_-eq [57] would amount to a revenue stream of roughly 165,000 €/a for the city.

The emissions reduction calculation in this study was based on several methodological assumptions that might have introduced a margin of error in the calculated results. Another limitation of this study was the lack of local data on dumpsite characteristics for baseline. These limitations cannot affect significantly the results gathered in this study. However, further research is needed to address these limitations and reduce the uncertainties in the calculation of gas emissions reduction potential of decentralized composting plants.

## 4. Conclusions

The objective of this study was to assess the material flows and the GHG emission reduction potential of a decentralized composting plant. The results show that

- On a dry weight basis, for every ton of waste treated, the facility can expect to obtain 360 kg of compost. In this context of waste composition and climate, it is important to note that 140 L of water are required to produce this amount of compost, i.e., 0.39 L per kilogram of compost. Access to and availability of this quantity of water could be problematic in arid zones. This study’s data provide a good estimate for the design and operation of future decentralized composting facilities.
- The nutrient content of the compost is important for agriculture when considering limited access to fertilizer markets whereby 1 metric ton of compost can substitute 37 kg of Urea. Heavy metal pollution in compost proved not relevant. Assessing the full benefits of compost for agriculture goes beyond only comparing nutrient values. For this purpose, compost utilization trials are currently being conducted to determine the optimal dose of compost for rice and tomato.
- Regarding the GHG emissions reduction potential of a decentralized composting plant, this study reveals that by composting, 87% of emissions from waste disposal can be avoided. These emissions avoided could be much higher if the avoidance of emissions from mineral fertilizer production and the carbon storage in soils due to compost utilization were counted. Therefore, decentralized composting can be an easy way for Sub-Saharan Africa governments to improve their waste management systems whilst reducing their GHG emissions in order to reach their intended nationally determined contributions.

## Figures and Tables

**Table 1:** Values used for baseline emissions calculation.

Parameter	Φ	fy [tCH_4_]	GWPCH_4_ [tCO_2_/tCH_4_]	OX	F	DOCf,y	MCFy
Values [27]	0.75	0	28	0	0.5	0.5	0.8

**Table 2:** Values used for project emissions calculation.

Parameter	EFCH_4_,y	GWPCH_4_ [tCO_2-eq_/tCH4]	EFN_2_O,y	GWPN_2_O [tCO_2-eq_/tN_2_O]
Values [27]	0.002	28	0.0002	298

**Table 3:** Inputs and outputs characteristics.

Parameters	PH	Water [%]	TS [%]	VS [%]	Ash [%]	C/N	N [g/kg TS]	P [g/kg TS]	K [g/kg TS]
Inputs	6.7 ± 0	41.6 ± 0.4	58.4 ± 0.4	47.8 ± 2	52.2 ± 2	34.0 ± 6.6	0.7 ± 0.2	2.7 ± 0.6	11.4 ± 0
Outputs	9.1 ± 0.4	12.6 ± 0.9	87.4 ± 0.9	36.9 ± 3.3	60.8 ± 7.2	11.3 ± 1	15.9 ± 1.3	5.2 ± 0.6	32.1 ± 1.5

**Table 4:** Compost heavy metal content in comparison to selected European standards (in mg/kg TS).

Parameters	Cd	Cr	Ni	Pb	Cu	Zn	Hg
France ^1^	3.0	120	60	180	300	600	2.0
Germany (class I compost)	1	70	35	100	70	300	0.7
Germany (class II compost) ^2^	1.5	100	50	150	100	300	1
Tiassalé	0.4 ± 0.2	20 ± 16.7	7.9 ± 0.1	18.7 ± 0.4	25.4 ± 0.9	152 ± 14.1	0.2 ± 0

^1^ French association of normalizations compost standards: NF U44-051 [14]. ^2^ Germany biowaste ordinance: compost application rates within a 3 year period: class I -30 t dry matter per ha; class II: 20 t dry matter per ha [52].

**Table 5:** Waste composition (fresh waste) and characteristics used for baseline calculation.

Waste Components	Food Waste	Garden Waste	Paper/Cardboard	Wood	Textiles	Inorganics
Proportion (% ww)	89.3	5.2	4.1	0.2	0.2	1.0
Wj,x (t)	53.04	3.1	2.4	0.1	0.1	0.6
DOCj (%) [39]	15	20	40	43	24	-
kj [39]	0.4	0.17	0.07	0.035	0.07	-

ww = wet weight; DOCj = Fraction of degradable organic carbon in the waste type j; Kj = Decay rate; Wj,x = Amount of solid waste type j disposed or prevented from disposal in the SWDS.

**Table 6:** Emissions reduction from CH_4_ and N_2_O given as CO_2_ equivalent.

	Baseline Emissions	Project Emissions	Emission Reduction
Quantity (tCO_2_-eq)	52.4	6.9	45.5
